# Knowledge, Attitudes, and Behaviors Regarding Sexually Transmitted Infections Among Romanian Medical Students: A Cross-Sectional Study

**DOI:** 10.3390/healthcare13101120

**Published:** 2025-05-12

**Authors:** Roxana-Denisa Capraș, Teodora Telecan, Răzvan Crețeanu, Carmen-Bianca Crivii, Alexandru-Florin Badea, Ariana-Anamaria Cordoș, Diana Roman-Pepine, Carmen-Maria Micu

**Affiliations:** 1 Department of Anatomy and Embryology, “Iuliu Hatieganu” University of Medicine and Pharmacy, 400012 Cluj-Napoca, Romania; capras.roxana@umfcluj.ro (R.-D.C.); creteanurazvandrc@yahoo.com (R.C.); bianca.crivii@umfcluj.ro (C.-B.C.); afbadea84@gmail.com (A.-F.B.); diana.pepine@yahoo.com (D.R.-P.); carmenmmicu@yahoo.com (C.-M.M.); 2 Department of Surgery-Practical Abilities, “Iuliu Hațieganu” University of Medicine and Pharmacy, 23 Marinescu Street, 400337 Cluj-Napoca, Romania; ariana.cordos@gmail.com

**Keywords:** reproductive health, medical students, sexually transmitted diseases, questionnaires

## Abstract

**Introduction:** Sexual and reproductive health is a critical aspect of medical education, yet significant knowledge gaps persist even among future healthcare professionals. This study aimed to evaluate the knowledge, behaviors, and attitudes of Romanian medical students regarding sexually transmitted infections (STIs) and contraceptive practices. **Materials and Methods:** A cross-sectional observational study was conducted on 510 undergraduate medical students, using a validated 30-item online questionnaire assessing socio-demographic data, sexual behaviors, STI knowledge, and attitudes towards sexual health. Descriptive statistics, chi-square, and Spearman correlation tests were used to analyze the data. **Results:** Participants demonstrated a good understanding of common STI pathogens and transmission routes, with 99.02% identifying unprotected vaginal intercourse as a risk factor. However, only 58.82% correctly identified Chlamydia trachomatis, and awareness of less common pathogens remained low. Long-term complications such as infertility (85.29%) and cervical cancer (87.25%) were well recognized, although misconceptions about STI severity persisted, with over 40% believing that STIs are not dangerous because they are treatable. Male students reported earlier sexual debut and more frequent high-risk behaviors, while females were more likely to consult specialist doctors and receive HPV vaccination. The level of knowledge correlated positively with parental education and faculty program. **Conclusions:** Despite generally high awareness of STI-related topics, considerable gaps and misconceptions persist among Romanian medical students. These findings highlight the need for comprehensive, structured sexual health education integrated into medical curricula to ensure future healthcare providers are well-equipped to promote public health.

## 1. Introduction

Sexual and reproductive education is a fundamental pillar of public health, playing a crucial role in preventing unplanned pregnancies and sexually transmitted infections (STIs). According to the World Health Organization, over one million people contract an STI daily, with individuals under 25 being the most vulnerable, as up to 32% of the teenage and young adult population present at least one STI, while 28.9% report no contraceptive use [1]. However, significant gaps in knowledge about contraception and STI prevention are still present, thus underlining the need for more comprehensive and targeted educational interventions [2].

Despite the increased availability of information, many individuals lack comprehensive knowledge about these subjects, often due to embarrassment or fear, which prevents them from seeking professional advice or engaging in informed discussions. This pattern is observed even among medical students and future health professionals. A study conducted on 1129 undergraduate nursing and physiotherapy students reported that only 23.1% of them received sexual education at school or during an educational talk, while 31.1% would refer to healthcare personnel for STI prevention information and means [3]. Another study conducted on 400 s- and fifth-year medical students reported that 34.7% of them received reproductive education as part of their university curriculum, 18.3% from attending extracurricular educational programs, while the rest resorted to family and friends [4].

Furthermore, additional studies reveal the complexity of sexual health knowledge and practices among university students. Despite a generally high level of awareness, there are evident gaps in the implementation of safe practices. A study conducted by Nzoputam et al. [5] reported that, although the interviewed students showed a high level of awareness (95.3%) and good knowledge (83.1%) about STIs, the prevalence of these conditions remains concerning, reaching 27.7%.

To date, few studies debating the subject of reproductive health and sexual behavior among medical students from Eastern European universities have been published. Subotic et al. [6] published a paper targeting 1237 volunteers from four undergraduate institutions in Serbia. The study showed that only 29.1% could name at least 4 STIs, 26% had knowledge about certain vaccines against some STIs, while less than half were aware of the link between Human Papilloma Virus (HPV) infection and cervical cancer. However, particularly for Romania, the education and opinions of undergraduate medical students concerning STIs, prevention methods, and resources have not been investigated.

The aim of the current study is to evaluate the reproductive health knowledge and sexual behaviors of Romanian medical students by developing and validating an online questionnaire form targeting these topics.

## 2. Materials and Methods

### 2.1. Study Design and Target Population

This study was conducted as an observational, cross-sectional cohort study. The target population included undergraduate students enrolled in one of the six years of study of the “Iuliu-Hațieganu” University of Medicine and Pharmacy study programs (Cluj-Napoca, Romania), between May and December of 2024.

The protocol was evaluated and approved by the local Ethics Committee (No. 66/19 April 2024, issued by the University of Medicine and Pharmacy “Iuliu Hațieganu”, Cluj-Napoca, Romania).

### 2.2. Questionnaire Development and Validation

**Figure 1:**
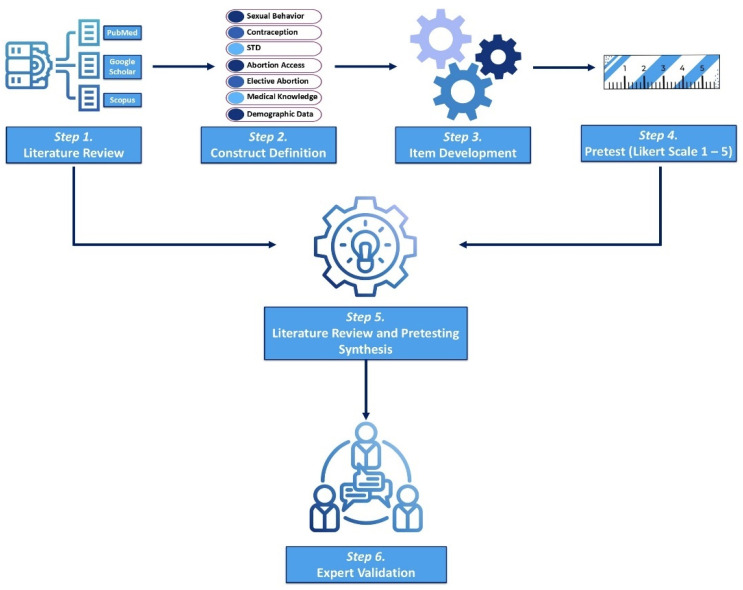
Graphical representation of the questionnaire development process.

The questionnaire was developed based on a 6-step process, which included (1) an extensive literature review, (2) construct definition, (3) item drafting, (4) pilot testing on a sample group, (5) revision based on participant feedback, and (6) validation by an expert panel. These steps are graphically represented in Figure 1.

The questionnaire comprised 30 questions, divided into 4 subsections:

1. Socio-demographic characteristics—This section collected general background information such as age, gender, year of study, marital and civil status, parental education level, and place of origin;
2. Sexual behavior—This part explored participants’ sexual history, including age of sexual debut, number of sexual partners, history of sexually transmitted infections, and behaviors related to alcohol or drug use during sexual activity;
3. Knowledge of sexually transmitted diseases—This section evaluated participants’ understanding of sexually transmitted infections, including their identification, modes of transmission, symptoms, and potential short- and long-term complications;
4. Attitudes towards sexually transmitted diseases—This section examined participants’ beliefs, opinions, and perceptions related to sexual health and preventive practices, using both Likert scale items and multiple-choice responses.

The questionnaire was designed in Romanian and subsequently translated into English for publication purposes (Table S1). The instrument was electronically distributed for validation to a sample of 70 medical students. Internal reliability was assessed using Cronbach’s Alpha coefficient, which reached a value of 0.99, indicating high internal consistency.

### 2.3. Data Collection

The questionnaire was distributed electronically using Google Forms (Google Inc., Mountain View, CA, USA) through the internal university network and was accompanied by an introductory letter, a self-assessment tool, and an informed consent to be agreed upon prior to filling in the questionnaire. All answers were given anonymously.

### 2.4. Statistical Analysis

Descriptive statistics were used to summarize the sociodemographic characteristics of the participants, sexual behaviors, knowledge, and attitudes towards sexually transmitted infections. Categorical variables were expressed as absolute frequencies (number) and relative frequencies (percentage). The association between categorical variables was assessed using the Chi-square (χ^2^) or Fisher’s test. The relationship between sociodemographic factors and participants’ knowledge, attitudes, and behaviors related to STIs was explored through Spearman’s rank correlation coefficients (Spearman’s rho). A *p*-value of less than 0.05 was considered statistically significant.

## 3. Results

### 3.1. General Characteristics of the Study Group

**Figure 2:**
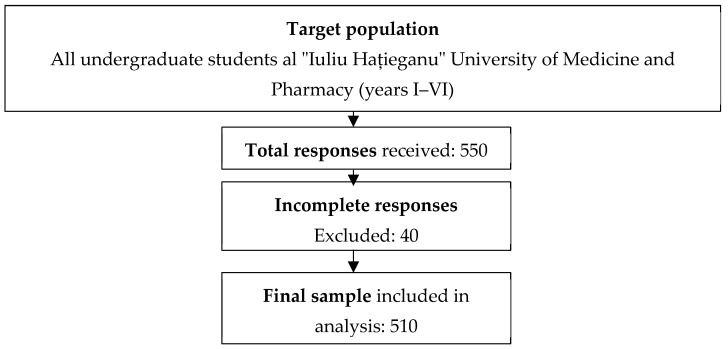
Flow diagram of sample selection from target population.

A total of 550 questionnaire responses were obtained, out of which 40 were excluded due to incomplete data, thus yielding a final analytical sample of 510 responses (Figure 2). The estimated completion time for the questionnaire was between 15 and 25 min. The majority of respondents were female, accounting for 77.45% of the study group. Likewise, 385 (75.49%) of the responders were enrolled in the General Medicine study program, 96 (18.82%) in Dentistry or Pharmacy, and 29 (0.57%) studied as Radiology and Medical Imaging technicians. The general characteristics of the study group are presented in Table 1.

Most participants were female, single, and from urban areas, with a balanced age distribution. Significant gender differences were observed in marital status, parental education, and year of study. Male students were predominantly in the first two years and more frequently enrolled in Dentistry or Radiology.

### 3.2. Sexual Behavior, Practices, and Personal Views

The analysis revealed that male participants reported an earlier sexual debut and a higher number of sexual partners in the last year compared to their female counterparts. HPV vaccination rates were significantly higher among female responders (46.84% versus 25.22%). Additionally, female students preferred consulting medical professionals such as gynecologists, urologists, or dermato-venereologists regarding STIs and contraception, whereas males were more inclined to seek advice from family doctors or online sources. Although not statistically significant, male students were more likely to report having had sexual intercourse under the influence of alcohol (48.7% vs. 43.5%) and drugs (10.4% vs. 8.9%), indicating a potential gender-related vulnerability in risk-taking behaviors. These differences are summarized in Table 2.

Our analysis revealed significant differences in students’ sexual behavior and attitudes depending on their year of study (Table 3). Students in the final years (V–VI) reported a higher prevalence of having had sexual intercourse (over 90%) compared to those in years I–II (around 67%), suggesting greater sexual experience with age and academic progression. Moreover, vaccination against HPV and engagement in discussions with healthcare professionals about sexually transmitted diseases (STDs) and contraception were significantly more frequent among senior students. This may reflect increased awareness and access to information as students advance in their studies, particularly given that sexual and reproductive health education is integrated into the medical curriculum in the later years. In contrast, early-year students were more likely to report using informal sources such as the internet or friends when seeking advice on sexual health, while older students predominantly consulted specialist doctors. These differences support the idea that formal education plays a key role in shaping responsible sexual behaviors and informed decision-making. Notably, sexual activity under the influence of alcohol was more common among students in the intermediate years (III–IV), while drug-influenced sexual encounters were rare across all groups and did not show significant variation. Overall, the findings highlight the need for structured and early inclusion of sexual and reproductive health education in the medical curriculum, as this may enhance knowledge and promote safer sexual practices from the first years of training. It is important to note that students in the early years of study (I–II) demonstrated greater interest and compliance with the survey, showing a higher response rate and greater engagement with the questionnaire. In contrast, students in the clinical years (V–VI), although politely invited several times, appeared less willing to participate or to complete the questionnaire thoroughly. This discrepancy in participation may reflect differences in workload, availability, or perceived relevance of the topic at different stages of training. It should be considered when interpreting the results, as it may have influenced the representativeness of the data, especially in the higher year groups.

The Spearman correlation analysis revealed a significant negative correlation between gender and HPV vaccination (ρ = −0.35, *p* < 0.001), indicating that male participants were less likely to be vaccinated against HPV compared to females. A positive correlation was observed between gender and the number of sexual partners in the last year (ρ = 0.28, *p* = 0.002), suggesting that male participants reported more sexual partners. Parental education was positively correlated with HPV vaccination (ρ = 0.25, *p* = 0.006). Students whose parents had higher education levels were more likely to be vaccinated. Age group showed a positive correlation with age at first sexual activity (ρ = 0.22, *p* = 0.015), indicating that older students tend to begin sexual activity later. Living in an urban environment showed a non-significant weak positive correlation with HPV vaccination (ρ = 0.15, *p* = 0.08). These findings are highlighted in Table 4.

The majority of participants disagreed with the statement that having multiple sexual partners does not increase the risk of STIs (64.90%). Most respondents (74.68% of females and 74.78% of males) recognized the importance of STI education in academic settings. While screening for STIs was widely perceived as beneficial, misconceptions persist—over 40% of respondents believed that STIs are not dangerous because they can be treated. Additionally, more than half (53.42% females and 52.17% males) acknowledged the potential lethality of untreated STIs, yet fear of contracting an STI was reported by less than half of the respondents (Table 5).

### 3.3. Students’ Knowledge of STIs and Associated Preventive Measures

The data shown in Table 6 highlight that the participants had varying levels of knowledge regarding pathogens responsible for sexually transmitted infections. While the majority recognized common agents such as Syphilis (83.92%), HPV (61.37%), and HIV (50.39%), awareness of other pathogens like Mycoplasma hominis (14.31%) and Ureaplasma urealyticum (12.94%) was significantly lower. Importantly, none of the participants managed to correctly identify all the listed STI pathogens, demonstrating considerable gaps in comprehensive knowledge about STIs.

Regarding the main transmission routes of sexually transmitted infections, the majority of respondents correctly identified them such as unprotected vaginal (99.02%) and anal intercourse (84.71%). However, fewer participants recognized vertical transmission (54.51%) or transmission through blood products (69.22%), with significant differences by gender (*p* = 0.014). Regarding symptoms, most respondents acknowledged abnormal discharge (90.98%), genital itching (94.12%), and ulcerative lesions (79.22%) as representative warning signs of STIs, although males were more likely to mention pain during intercourse (*p* = 0.022). Concerning complications, infertility (85.29%) and cervical cancer (87.25%) were widely recognized. Despite these results, no respondent identified all possible transmission routes, symptoms, and complications (Table 7).

The Spearman’s correlation analysis (Table 8) revealed significant associations between sociodemographic factors and the knowledge of transmission routes, symptoms, and STIs among undergraduate students. Gender showed a strong positive correlation with knowledge of penile cancer (ρ = +0.42, *p* = 0.00034), indicating that male participants were more aware of this condition. Study program type was significantly associated with knowledge of infertility as a long-term complication (ρ = +0.30, *p* = 0.004) and transmission through blood products (ρ = +0.26, *p* = 0.014). Parental education showed a positive correlation with knowledge of cervical cancer (ρ = +0.25, *p* = 0.007). Gender was also correlated with knowledge of pain during sexual intercourse as a symptom of STIs (ρ = +0.29, *p* = 0.022).

Lastly, when questioned about their view on the regular use of barrier contraceptive methods, most participants considered condoms to be an effective method of preventing STIs, although 64.31% recognized that condoms cannot prevent all infections. Approximately 41.18% completely agreed that condoms are the safest method for STI prevention. However, misconceptions remain: 5.22% of male respondents believed condoms are unnecessary during anal intercourse, and 7.83% of male students thought that condoms are not necessary if both partners are already infected. These findings highlight a generally positive perception of condom effectiveness; however, they also underline the need for improved education regarding their consistent use in all sexual practices. These data are presented in Table 9.

When asked about their reasons for not using condoms during sexual encounters, the participants cited several reasons, the most frequently mentioned one being the belief that condoms reduce sexual pleasure, reported by 42.16% of respondents. This perception was significantly more common among men (64.35%) compared to women (35.70%). Another common reason was complete trust in their sexual partner, mentioned by 39.75% of participants, with similar proportions among both sexes. Additionally, 8.10% stated they were ready to face the consequences of unprotected intercourse, a reason more frequently expressed by men (13.04%) than women (4.30%). Other reported reasons included embarrassment when purchasing condoms (11.90%) and difficulties in obtaining them (6.33%). A small percentage of participants (3.29%) mentioned being allergic to the materials used in condoms, without significant gender differences.

## 4. Discussion

The results obtained from this study provide a detailed perspective on the behaviors, knowledge, and attitudes of Romanian medical students regarding sexual health and the prevention of sexually transmitted infections. The data analysis highlights both positive aspects, such as a high level of awareness regarding certain preventive methods and pathogens, as well as significant deficiencies in key areas of reproductive health. Thus, this study facilitates a better understanding of the strengths, limitations, and opportunities for improving sexual education in Romanian academic settings.

The sample included a predominance of female respondents, accounting for 77.45% of the participants. This gender imbalance is reflective of the general demographics within medical universities in Romania and in similar European contexts, where women represent a substantial proportion of medical students. Similar findings have been reported in previous studies, suggesting a feminization trend in medical education worldwide [7]. As demonstrated by Pickel et al. [8], who analyzed gender trends in medicine and surgery over the past three decades, a significant increase in the number of women entering the medical field was observed, although certain surgical specialties remain male dominated.

Regarding HPV vaccination, female students had a significantly higher vaccination rate (46.84%) compared to their male counterparts (25.22%). This imbalance is often explained by the widespread perception that HPV vaccination is primarily aimed at females, due to its role in preventing cervical cancer. This issue seems to be persistent worldwide, as Zhang et al. reported that although 90.2% of students had heard of HPV, only 69.2% were aware of the HPV vaccine, and 55.9% would be willing to undergo the vaccination process [9].

Several publications targeting female undergraduate students highlight significant gaps in knowledge and uptake of cervical cancer prevention. A study conducted on 584 non-medical female students showed that only 40.5% of them were aware of cervical cancer, 35.6% had good knowledge, and just 0.9% had ever been screened, the main barrier being the lack of information [10]. A concerning aspect of these studies was the lack of awareness between HPV infection and cervical cancer, as the percentage of undergraduate students who are well informed in this matter varies from 19.5% to 52.5% [11,12].

In terms of HPV immunization, Romania faces a double challenge: financial and educational. The Romanian National Vaccination Program does not include this vaccine; thus, females aged 18 to 45 need to pay 50% of the full price, which for some socioeconomic subgroups might be an important financial burden [13]. From the educational perspective, parental education correlates positively with HPV vaccination rates, as shown in the current study. However, studies conducted by Romanian researchers show that, although parents have good knowledge of HPV infection and rely mostly on medical professionals for HPV-related information and recommendations, only 10.7% are actually vaccinated [13,14].

Regarding access to healthcare services, most participants of the study had previously discussed sexually transmitted infections and contraception issues with healthcare professionals. In case of a suspected STI, females predominantly preferred consulting a specialist doctor (76.46%), while males were more inclined to seek help from a family doctor (36.52%). The study conducted on 504 non-medical students published by Mcharo et al. [15] demonstrated that while 79% of the respondents had heard of sexually transmitted infections, only 54.3% could identify specific sources from which they had received information. The most commonly reported source was university-based lectures (45.5%), followed by media, books, the internet, and explicit websites (20.5%), family members (14.6%), and peers of similar age (8.8%). Alarmingly, only 8.2% of students reported having received information from healthcare professionals, such as doctors, nurses, or reproductive health clinics. These findings are in contrast with our study, where a large proportion of students reported having consulted authorized healthcare services.

In terms of the level of knowledge concerning pathogens responsible for sexual transmitted infections, the majority of respondents correctly identified common pathogens such as Treponema pallidum (syphilis) (83.92%), human papillomavirus (61.37%), and HIV (50.39%), awareness of other STI pathogens such as Neisseria gonorrhoeae and Chlamydia trachomatis was insufficient, with only 50% and 58.82% of respondents identifying them, respectively. These results are comparable to those reported in a 2024 French study, where only 48.2% and 44.5% of health sciences students correctly recognized *Chlamydia trachomatis* and *Neisseria gonorrhoeae*, respectively [16]. Gender differences were also observed. Male respondents demonstrated significantly higher awareness of Herpes simplex virus (75.65% vs. 64.30%) and HIV (94.78% vs. 36.20%), which may reflect greater exposure to prevention campaigns historically targeted toward male populations [17]. On the other hand, men were also more likely to associate Candida spp. (50.43% vs. 36.96%) with STIs, potentially indicating conceptual confusion between sexually transmitted infections and opportunistic fungal infections, highlighting the need for clearer educational messaging [18]. Students reported a high awareness of key transmission routes such as unprotected vaginal (99%) and anal intercourse (84.7%), as well as oral sex (80.8%). Most participants correctly identified common symptoms, including genital itching (94.1%), abnormal discharge (91%), and pain during intercourse (78.4%). Regarding long-term complications, infertility (85.3%) and cervical cancer (87.2%) were the most commonly recognized outcomes, though awareness of conditions like ectopic pregnancy (43.3%) or pelvic inflammatory disease (43.1%) was notably lower. These results are consistent with previously published papers. A study conducted by Dorji et al. [19] found that 53.2% of participants demonstrated good knowledge of sexually transmitted infections, with HIV/AIDS being the most commonly recognized infection (93.8%), while awareness of other STIs such as syphilis (31.1%) and hepatitis B (21.1%) remained low. Although the majority correctly identified unprotected sexual intercourse as a mode of transmission (86.9%), fewer participants recognized other transmission routes like sharing contaminated needles (53.4%) or mother-to-child transmission (38.7%). Additionally, only 60.1% associated cervical cancer as a possible complication of STIs, indicating a partial understanding of long-term consequences. These findings reinforce the idea that university students often lack comprehensive knowledge about STIs, while highlighting the importance of targeted sexual health education programs.

Another key point of the present study is the participants’ perception of the importance of sexual health education. While most participants recognized the importance of academic institutions addressing STI education (74.68% of females and 74.78% of males totally disagreed with the statement that such discussions are unnecessary), misconceptions regarding STI risks and severity still persist. Over 40% of respondents believed that STIs are “not dangerous because they can be treated”, which minimizes the risks associated with delayed or inadequate treatment, such as infertility, ectopic pregnancy, and increased HIV transmission risk [20].

In our study, 98.43% of participants recognized that condoms help prevent STIs, with 64.31% believing they offer partial protection and 34.12% believing they prevent all STIs. Only 1.57% denied their effectiveness. While 84.71% agreed that condoms are the safest method against STIs, misconceptions remain: 6.08% believed they are unnecessary during anal sex, and 6.47% if both partners are infected. These findings align with a 2023 Portuguese study by Santos, where 50.4% of students acknowledged condoms’ partial effectiveness and 43.6% believed in complete protection. The similarities suggest an overestimation of condom efficacy and highlight the need for improved educational efforts across European university settings [21].

The most frequently cited reason for not using condoms was reduced sexual pleasure (42.16%), especially among men (64.35%), consistent with findings from Santos et al. [21], where 45.9% of college students reported the same barrier. Trust in one’s partner was the second most common reason in both studies, highlighting a shared perception that emotional intimacy reduces perceived risk.

## 5. Limitations

This study presents a few limitations that should be acknowledged. Firstly, the cross-sectional design does not allow for the establishment of causal relationships between variables. Secondly, the data were self-reported, which may have introduced social desirability or recall biases, particularly regarding sensitive topics such as sexual behaviors and STI history. Thirdly, although the questionnaire was validated and distributed through institutional channels, the voluntary and anonymous nature of participation may have led to selection bias, with more health-conscious or informed individuals being more likely to respond. Additionally, the predominance of female respondents reflects the general demographic structure of medical faculties but may limit the generalizability of the findings to male students. Lastly, the study was conducted in a single medical university in Romania, which may restrict the applicability of the results to other academic institutions or regions.

## 6. Perspectives and Future Directions

Given the persistence of misconceptions regarding sexually transmitted infections (STIs) observed in our study, there is a clear need for earlier and more structured sexual health education in medical training. In Romania, STI-related content is formally introduced only in the fifth or sixth year, primarily through dermatology and gynecology modules. However, these topics are often addressed superficially, with limited clinical application unless students later specialize in these fields. Consequently, many students may complete their studies without sufficient exposure to the complexities of STI prevention, diagnosis, and counseling. This is even more concerning for students in non-medical faculties, who typically receive no formal education on sexual and reproductive health.

To address this gap, we advocate for the integration of sexual and reproductive health education from the first years of medical training. Interactive, case-based learning sessions, peer-led workshops, and collaboration with specialists in infectious diseases and gynecology could enhance knowledge and engagement. Pre- and post-intervention evaluations should be incorporated into future studies to assess the impact of such educational reforms. Moreover, using digital tools and partnerships with student organizations may facilitate broader dissemination and reach underserved student groups.

The results of this study reveal important gaps in knowledge and significant gender-based differences in sexual health attitudes among medical students, which underscore the need for curriculum enhancement. To address these challenges, medical education should integrate more focused training on reproductive immunology, STI prevention, and the public health implications of HPV and related cancers. Recent literature, including Hamoud et al. [22] and Cibula [23], highlights the expanding role of immunotherapy in managing uterine and HPV-associated cancers, offering new avenues for education and prevention strategies. In parallel, Prize et al. [24] emphasize that both residents and medical specialists express a need for improved and updated training in reproductive and sexual health. Embedding these insights into the curriculum could bridge the knowledge-practice gap and prepare future physicians to better address sexual health issues in diverse patient populations.

Finally, the visual and tabular elements are well-presented, and the Supplementary File enhances the transparency of the validation process. Ethical considerations and data availability are appropriately addressed, indicating adherence to responsible research practices.

## 7. Conclusions

This study provides a comprehensive evaluation of the sexual health knowledge, behaviors, and attitudes among Romanian medical undergraduate students. Despite relatively high levels of awareness regarding certain aspects of sexual and reproductive health, significant gaps remain, particularly in the understanding of STI transmission, prevention, and the full range of contraceptive options available.

## Figures and Tables

**Table 1:** Sociodemographic characteristics of the study group.

Variables	Female (395)	Male (115)	Total (510)	*p*-Value
	No (%)	No (%)	No (%)	
**Age (years)**				0.650
18–20	209 (40.98)	55 (10.78)	264 (51.76)	
21–24	142 (27.84)	44 (8.63)	186 (36.47)	
>24	44 (8.63)	16 (3.14)	60 (11.76)	
**Marital status**				* <0.001
Single	209 (40.98)	62 (12.16)	271 (53.14)	
In a relationship	142 (27.84)	50 (9.80)	192 (37.65)	
Married	44 (8.63)	1 (0.20)	45 (8.82)	
**Original environment**				0.983
Urban	298 (58.43)	86 (16.86)	384 (75.29)	
Rural	97 (19.02)	29 (5.69)	126 (24.71)	
**Parents’ education**				0.024
<High School	14 (2.75)	10 (1.96)	24 (3.88)	
High School	75 (14.71)	14 (2.75)	89 (18.64)	
Higher Education	306 (60.00)	91 (17.84)	397 (77.09)	
**Faculty**				* 0.019
Medicine	308 (60.39)	77 (15.10)	385 (75.49)	
Dental Medicine/Pharmacy	64 (12.55)	32 (6.27)	96 (18.82)	
Radiology and Medical Imaging	23 (4.51)	6 (1.18)	29 (0.57)	
**Year of study**				* 0.002
I–II	274 (69.37)	93 (80.87)	367 (71.96)	
III–IV	66 (16.71)	18 (15.65)	84 (16.47)	
V–VI	55 (13.92)	4 (3.48)	59 (11.57)	

Chi-square test and * Fisher’s exact test.

**Table 2:** Sexual behavior of the included respondents.

Variables	Female (395)	Male (115)	Total (510)	*p*-Value
	No (%)	No (%)	No (%)	
**Have you ever had sexual intercourse with penetration?**				* 0.70
Yes	282 (71.39)	85 (73.91)	367 (71.96)	
No	105 (26.58)	27 (23.48)	132 (25.88)	
Prefer not to answer	8 (2.03)	3 (2.61)	11 (2.16)	
**At what age did you start your sexual activity?**				* 0.1
<18 years	163 (41.27)	62 (53.91)	225 (44.12)	
≥18 years	133 (33.67)	30 (26.09)	163 (31.96)	
I have not started my sexual life yet	89 (22.53)	22 (19.13)	111 (21.76)	
Prefer not to disclose	10 (2.53)	1 (0.87)	11 (2.16)	
**The number of sexual partners in the last year**				* 0.0051
0	119 (30.13)	30 (26.09)	149 (29.22)	
1	205 (51.90)	47 (40.87)	252 (49.41)	
≥2 sexual partners	64 (16.20)	35 (30.43)	99 (19.41)	
Prefer not to disclose	7 (1.77)	3 (2.61)	10 (1.96)	
**Have you ever had sexual relations under the influence of alcohol?**				* 0.063
Yes	172 (43.54)	56 (48.70)	228 (44.71)	
No	221 (55.95)	56 (48.70)	277 (54.31)	
I don’t remember	2 (0.51)	3 (2.61)	5 (0.98)	
**Have you ever had sexual relations under the influence of prohibited substances (e.g., drugs)?**				* 0.18
Yes	35 (8.86)	12 (10.43)	47 (9.22)	
No	360 (91.14)	102 (88.70)	462 (90.59)	
I do not remember	0 (0)	1 (0.87)	1 (0.20)	
**Have you been vaccinated against human papillomavirus (HPV)?**				<0.001
Yes	185 (46.84)	29 (25.22)	216 (42.35)	
No	172 (43.54)	64 (55.65)	238 (46.67)	
I don’t know	38 (9.62)	22 (19.13)	61 (11.96)	
**Have you ever contracted a sexually transmitted infection?**				0.16
Yes	23 (5.82)	9 (7.83)	32 (6.27)	
No	361 (91.39)	99 (86.09)	460 (90.20)	
I do not know	11 (2.78)	7 (6.09)	18 (3.53)	
**Have you ever discussed with healthcare professionals about sexually transmitted infections and contraception?**				0.53
Yes	279 (70.63)	80 (69.57)	359 (70.39)	
No	104 (26.33)	29 (25.22)	133 (26.08)	
I don’t remember	12 (3.04)	6 (5.22)	18 (3.53)	
**In case of contracting a sexually transmitted infection, who would you prefer to consult?**				* <0.001
Pharmacist	2 (0.51)	2 (1.74)	4 (0.78)	
Family doctor	65 (16.46)	42 (36.52)	107 (20.98)	
Specialist doctor (gynecologist/urologist/dermatologist)	302 (76.46)	66 (57.39)	368 (72.16)	
Prefer to search for information on the Internet	18 (4.56)	4 (3.48)	22 (4.31)	
Prefer to seek advice from friends/colleagues	8 (2.03)	1 (0.87)	9 (1.76)	
**If you want advice on contraception, who would you prefer to consult?**				<0.001
I discuss with my parents/family	32 (8.10)	15 (13.04)	47 (9.22)	
I discuss with friends/colleagues	39 (9.87)	18 (15.65)	57 (11.18)	
I consult my family doctor	31 (7.85)	23 (20.00)	54 (10.59)	
I consult a specialist doctor	225 (56.96)	29 (25.22)	254 (49.80)	
I prefer to search for information on the Internet	68 (17.22)	30 (26.09)	98 (19.22)	

Chi-square test and * Fisher’s exact test.

**Table 3:** Distribution of sexual health-related behaviors and choices among medical students by study year.

	Year of Study			*p*-Value
Variables	I–II No = 367 (%)	III–IV No = 84 (%)	V–VI No = 59 (%)	
**Sexual intercourse**				<0.001
No	112 (30.5)	17 (20.2)	4 (6.8)	
Prefer not to answer	10 (2.7)	0 (0.0)	0 (0.0)	
Yes	245 (66.8)	67 (79.8)	55 (93.2)	
**Sexual Debut Age**				
<18 years	167 (45.5)	30 (35.7)	28 (47.5)	<0.001
I have not started my sexual life yet	93 (25.3)	15 (17.9)	4 (6.8)	
Prefer not to answer	11 (3.0)	0 (0.0)	0 (0.0)	
≥18 years	96 (26.2)	39 (46.4)	27 (45.8)	
**HPV Vaccinated**				0.003
I do not know	53 (14.4)	4 (4.8)	2 (3.4)	
No	165 (45.0)	49 (58.3)	24 (40.7)	
Yes	149 (40.6)	31 (36.9)	33 (55.9)	
**Discussed STDs**				
I don’t remember	14 (3.8)	1 (1.2)	1 (1.7)	0.0034
No	106 (28.9)	21 (25.0)	7 (11.9)	
Yes	247 (67.3)	62 (73.8)	51 (86.4)	
**Had STD**				
I don’t know	14 (3.8)	3 (3.6)	0 (0.0)	0.0046
No	334 (91.0)	78 (92.9)	49 (83.1)	
Yes	19 (5.2)	3 (3.6)	10 (16.9)	
**Whom Address STD**				
Family doctor	68 (18.5)	26 (31.0)	14 (23.7)	<0.001
I prefer to seek advice from friends/colleagues	4 (0.8)	0 (0.0)	0 (0.0)	
Pharmacist	3 (0.8)	0 (0.0)	1 (1.7)	
Prefer to search for information on the Internet	22 (6.0)	4 (4.8)	1 (1.7)	
gynecologist/urologist/dermatologist	270 (73.6)	54 (64.3)	43 (72.9)	
**Sex alcohol**				
I don’t remember	4 (1.1)	0 (0.0)	1 (1.7)	<0.001
No	218 (59.4)	42 (50.0)	18 (30.5)	
Yes	145 (39.5)	42 (50.0)	40 (67.8)	
**Sex drugs**				
I don’t remember	0 (0.0)	1 (1.2)	0 (0.0)	0.026
No	339 (92.4)	73 (86.9)	49 (83.1)	
Yes	28 (7.6)	10 (11.9)	10 (16.9)	
**Whom Address Contraception**				
gynecologist/urologist	175 (47.7)	41 (48.8)	38 (64.4)	0.13
I consult my family doctor	10 (2.7)	0 (0.0)	0 (0.0)	
I discuss with friends/colleagues	26 (7.1)	9 (10.7)	4 (6.8)	
I discuss with my parents/family	79 (21.5)	19 (22.6)	12 (20.3)	

Chi-square test.

**Table 4:** Spearman’s correlation between sociodemographic factors and sexual behaviors amongst undergraduate students.

Variable 1	Variable 2	Spearman Rho	*p*-Value
Sex	HPV Vaccination	−0.35	<0.001
Sex	Number of sexual partners	+0.28	0.002
Parents’ education	HPV Vaccination	+0.25	0.006
Faculty	Discussed with Specialist	+0.30	0.001
Age group	Age of sexual debut	+0.22	0.015
Marital status	Number of sexual partners	−0.18	0.042
Environment (Urban)	HPV Vaccination	+0.15	0.08

**Table 5:** Participants’ views on sexual behavior, education, and the prevention of STIs.

	Female (395)	Male (115)	Total (510)	*p*-Value
	No (%)	No (%)	No (%)	
**Having multiple sexual partners does not mean an increased risk of contracting a sexually transmitted infection.**				0.084
Totally agree	10 (2.53)	8 (6.96)	18 (3.53)	
Agree	32 (8.10)	12 (10.43)	44 (8.63)	
Disagree	91 (23.04)	26 (22.61)	117 (22.94)	
Totally disagree	262 (66.33)	69 (60.00)	331 (64.90)	
**Is not necessary for academic institutions to discuss issues related to sexually transmitted infections with students.**				0.031
Totally agree	6 (1.52)	2 (1.74)	91 (17.84)	
Agree	7 (1.77)	6 (5.22)	177 (34.71)	
Disagree	87 (22.03)	21 (18.26)	150 (29.41)	
Totally disagree	295 (74.68)	86 (74.78)	92 (18.04)	
**Legal repression of prostitution can reduce the spread of sexually transmitted infections.**				0.049
Totally agree	66 (16.71)	25 (21.74)	378 (74.12)	
Agree	143 (36.20)	34 (29.57)	112 (21.96)	
Disagree	120 (30.38)	30 (26.09)	15 (2.94)	
Totally disagree	66 (16.71)	26 (22.61)	5 (0.98)	
**Screening for sexually transmitted infections is beneficial.**				* 0.077
Totally agree	290 (73.42)	88 (76.52)	5 (0.98)	
Agree	90 (22.78)	22 (19.13)	19 (3.73)	
Disagree	11 (2.78)	4 (3.48)	210 (41.18)	
Totally disagree	4 (1.01)	1 (0.87)	276 (54.12)	
**Transmitted infections are not dangerous because they can be treated.**				* 0.062
Totally agree	4 (1.01)	1 (0.87)	271 (53.14)	
Agree	14 (3.54)	5 (4.35)	208 (40.78)	
Disagree	168 (42.53)	42 (36.52)	23 (4.51)	
Totally disagree	209 (52.91)	67 (58.26)	8 (1.57)	
**Sexually transmitted infections can cause the death of the patient if not treated.**				* 0.414
Totally agree	211 (53.42)	60 (52.17)	180 (35.29)	
Agree	162 (41.01)	46 (40.00)	212 (41.57)	
Disagree	18 (4.56)	5 (4.35)	87 (17.06)	
Totally disagree	4 (1.01)	4 (3.48)	36 (7.06)	
**I am afraid of contracting a sexually transmitted infection.**				0.223
Totally agree	140 (35.44)	37 (32.17)	215 (42.16)	
Agree	158 (40.00)	52 (45.22)	25 (4.90)	
Disagree	69 (17.47)	18 (15.65)	47 (9.22)	
Totally disagree	28 (7.09)	8 (6.96)	13 (2.55)	

Chi-square test and * Fisher’s exact test.

**Table 6:** Gender-based knowledge of pathogens responsible for STIs.

	Female (395)	Male (115)	Total (510)	*p*-Value
	No (%)	No (%)	No (%)	
**Select from the list below the pathogens that can cause sexually transmitted infections (STIs):**				
*Gardnerella vaginalis*	123 (31.14)	46 (40.00)	173 (33.92)	0.096
*Ureaplasma urealyticum*	45 (11.39)	19 (16.52)	66 (12.94)	0.193
*Mycoplasma hominis*	45 (11.39)	26 (22.61)	73 (14.31)	0.0036
*Trichomonas vaginalis*	166 (42.03)	43 (37.39)	212 (41.57)	0.434
*Herpes simplex*	254 (64.30)	87 (75.65)	344 (67.45)	0.0305
*Sifilis*	323 (81.77)	101 (87.83)	428 (83.92)	0.166
*Fungal infections (* *e.g., Candida spp.)*	146 (36.96)	58 (50.43)	208 (40.78)	0.0128
*Neisseria Gonorrhoeae*	198 (50.13)	53 (46.09)	255 (50.00)	0.511
*Chlamydia trachomatis*	225 (56.96)	72 (62.61)	300 (58.82)	0.330
*Papilovirusul uman (HPV)*	231 (58.48)	77 (66.96)	313 (61.37)	0.127
*Molluscum contagiosum*	13 (3.29)	5 (4.35)	19 (3.73)	0.800
*Actinomices*	3 (0.76)	0 (0)	3 (0.59)	* 1.000
*Scabies*	21 (5.32)	8 (6.96)	29 (5.69)	0.660
*Pediculosis*	6 (1.52)	0 (0)	6 (1.18)	* 0.345
*Hepatitis B virus*	123 (31.14)	34 (29.57)	161 (31.57)	0.836
*Hepatitis C virus*	24 (6.08)	7 (6.09)	35 (6.86)	1.000
*HIV (human immunodeficiency virus)*	143 (36.20)	109 (94.78)	257 (50.39)	<0.001
I do not know	6 (1.52)	1 (0.87)	7 (1.37)	* 1.000

Chi-square test and * Fisher’s exact test.

**Table 7:** Knowledge and perceptions regarding STIs transmission routes, symptoms, and complications.

	Female (395)	Male (115)	Total (510)	*p*-Value
**According to you, what are the transmission routes of STIs?**	No (%)	No (%)	No (%)	
Unprotected vaginal intercourse	387 (97.97)	113 (98.26)	505 (99.02)	1.000
Unprotected anal intercourse	328 (83.04)	100 (88.50)	432 (84.71)	0.388
Oral sex	315 (81.40)	94 (94.00)	412 (80.78)	0.735
Transmission from mother to child during vaginal childbirth	207 (63.11)	69 (73.40)	278 (54.51)	0.183
Transmission through blood or other infected biological products	259 (82.22)	90 (78.26)	353 (69.22)	0.014
I do not know	7 (3.38)	0 (0)	7 (1.37)	* 0.358
**Among the following symptoms, which do you think may be present in sexually transmitted infections?**				
Pelvic pain	275 (69.62)	81 (70.43)	359 (70.39)	0.958
Pain during sexual intercourse	298 (75.44)	99 (86.09)	400 (78.43)	0.022
Bleeding during sexual intercourse	252 (63.80)	74 (64.35)	329 (64.51)	1.000
Abnormal vaginal or urethral discharge	359 (90.89)	100 (86.96)	464 (90.98)	0.809
Itching in the genital or anal areas	368 (93.16)	108 (93.91)	480 (94.12)	0.944
Burning sensations in the genital areas	362 (91.65)	102 (88.70)	469 (91.96)	0.431
Ulcerative lesions in the genital areas	308 (77.97)	92 (80.00)	404 (79.22)	0.737
I do not know	6 (1.52)	0 (0)	6 (1.18)	* 0.345
**In your opinion, what are the long-term complications of sexually transmitted infections?**				
Infertility	331 (83.80)	101 (87.83)	435 (85.29)	0.363
Cervical cancer	343 (86.84)	100 (86.96)	445 (87.25)	1.000
Penile cancer	216 (54.68)	85 (73.91)	303 (59.41)	<0.001
Pelvic inflammatory disease	162 (41.01)	56 (48.70)	220 (43.14)	0.174
Chronic pelvic pain	248 (62.78)	83 (72.17)	332 (65.10)	0.081
Increased risk of ectopic pregnancy	162 (41.01)	57 (49.57)	221 (43.33)	0.128
I do not know	8 (2.03)	1 (0.87)	10 (1.96)	* 0.691

Chi-square test and * Fisher’s exact test.

**Table 8:** Spearman’s correlation between sociodemographic factors and knowledge of STIs transmission routes, symptoms, and complications, amongst undergraduate students.

Variable 1	Variable 2	Spearman Rho	*p*-Value
Sex	Knowledge of Penile Cancer	+0.42	<0.001
Faculty	Knowledge of Infertility	+0.30	0.004
Parents’ Education	Knowledge of Cervical Cancer	+0.25	0.007
Age Group	Knowledge of Chronic Pelvic Pain	+0.22	0.015
Marital Status	Knowledge of Ectopic Pregnancy Risk	+0.18	0.042
Sex	Knowledge of Pain During Intercourse	+0.29	0.022
Faculty	Knowledge of Transmission Through Blood	+0.26	0.014

**Table 9:** Participants’ perceptions regarding condom use in the prevention of STIs.

	Female (395)	Male (115)	Total (510)	*p* Value
	No (%)	No (%)	No (%)	
**In your opinion, can condoms prevent the occurrence of STIs?**				* 0.352
Yes, but it cannot prevent all STIs	258 (65.32)	70 (60.87)	328 (64.31)	
Yes, it can prevent all STIs	130 (32.91)	44 (38.26)	174 (34.12)	
No	7 (1.77)	1 (0.87)	8 (1.57)	
**In your opinion, the condom is the safest method to protect against sexually transmitted infections.**				0.422
Totally agree	157 (39.75)	53 (46.09)	210 (41.18)	
Agree	176 (44.56)	46 (40.00)	222 (43.53)	
Disagree	48 (12.15)	11 (9.57)	59 (11.57)	
Totally disagree	14 (3.54)	5 (4.35)	19 (3.73)	
**Do you think the use of a condom is unnecessary during anal intercourse?**				0.091
Totally agree	9 (2.28)	6 (5.22)	15 (2.94)	
Agree	8 (2.03)	8 (6.96)	16 (3.14)	
Disagree	87 (22.03)	28 (24.35)	115 (22.55)	
Totally disagree	291 (73.67)	73 (63.48)	364 (71.37)	
**If both partners have a sexually transmitted infection, I do not think the use of a condom is necessary during sexual intercourse.**				* 0.184
Totally agree	15 (3.80)	9 (7.83)	24 (4.71)	
Agree	6 (1.52)	3 (2.61)	9 (1.76)	
Disagree	73 (18.48)	17 (14.78)	90 (17.65)	
Totally disagree	301 (76.20)	86 (74.78)	387 (75.88)	

Chi-square test and * Fisher’s exact test.

## Data Availability

The original contributions presented in this study are included in the article. Further inquiries can be directed to the corresponding author.

## Supplementary Materials

The following supporting information can be downloaded at https://www.mdpi.com/article/10.3390/healthcare13101120/s1, Table S1: Questionnaire items and Likert scale results obtained by each question in the validation phase.
