# Supplementary material for: Knowledge, Attitudes, and Behaviors Regarding Sexually Transmitted Infections Among Romanian Medical Students: A Cross-Sectional Study
Source: Healthcare (Basel). 2025 May 12;13(10):1120. doi: 10.3390/healthcare13101120 (PMC12110783; doi:10.3390/healthcare13101120)
Supplement: Supplementary file 1 [file healthcare-13-01120-s001.zip › healthcare-3615190-supplementary.pdf]

Item of the instrument	M±SD
1. Have you ever had sexual intercourse with penetration?	4.56±0.75
2. At what age did you start your sexual activity?	4.37±1.07
3. Have you been vaccinated against the Human Papillomavirus (HPV)?	4.68±0.59
4. Specify the number of sexual partners in the last year.	4.28±1.11
5. Have you ever discussed sexually transmitted infections and contraception with healthcare professionals?	4.56±0.8
6. Have you ever contracted a sexually transmitted disease?	4.31±1.06
7. In case of contracting a sexually transmitted infection, who would you prefer to consult?	4.65±0.54
8. Have you ever had sexual relations under the influence of alcohol?	4.4±0.83
9. Have you ever had sexual relations under the influence of prohibited substances (e.g., drugs)?	4.43±0.87
10. If you want advice on contraception, who would you prefer to consult?	4.53±0.62
11. Select the contraceptive methods you have heard of:	4.62±0.60
12. Select the contraception methods you currently use:	4.68±0.53
13. Do you consider access to available contraception methods easy?	4.56±0.66
14. Select from the list below the pathogens that can cause sexually transmitted infections (STIs):	4.62±0.60
15. According to you, what are the transmission routes of STIs?	4.65±0.60
16. In your opinion, can condoms prevent the occurrence of STIs?	4.65±0.54
17. Among the following symptoms, which do you think may be present in sexually transmitted infections?	4.62±0.60
18. In your opinion, what are the long-term complications of sexually transmitted infections?	4.62±0.60
19. In your opinion, the condom is the safest method to protect against sexually transmitted infections.	4.62±0.55
20. Do you think the use of a condom is unnecessary during anal intercourse?	4.43±1.04
21. If both partners are carriers of a sexually transmitted disease, I do not consider it necessary to use a condom.	4.46±1.04
22. Having multiple sexual partners does not mean an increased risk of contracting a sexually transmitted infection.	4.15±1.41
23. It is not necessary for academic institutions to discuss sexually transmitted diseases with students.	4.31±1.14
24. Legal repression of prostitution can reduce the spread of sexually transmitted infections	4.43±1.01
25. Screening for sexually transmitted infections is beneficial.	4.71±0.52
26. STDs are not dangerous because they can be treated.	4.18±1.33
27. STDs can cause the death of the patient if untreated.	4.56±0.84
28. I am afraid about contracting a sexually transmitted disease.	4.43±0.87
29. In the case of unprotected sexual intercourse, what is your main concern?	4.56±0.71
30. The reasons why you prefer not to use a condom during sexual intercourse...	4.43±0.98
31. Select your gender	4.62±0.75
32. Select your age group	4.71±0.58
33. Marital status	4.43±0.91
34. Select the faculty you are studying at	4.59±0.79
35. If you are a medical student, choose your specialization	4.68±0.64

36. Select your year of study	4.68±0.64
37. Select de education level of your parents	4.5±0.87
38. Choose the original environment	4.62±0.87

Supplementary Table S1. Questionnaire items and Likert scale result obtained by each question in the validation phase.
M = mean; **SD =** Standard Deviation
